# IL-32 promotes breast cancer cell growth and invasiveness

**DOI:** 10.3892/ol.2014.2641

**Published:** 2014-10-27

**Authors:** SHOUMAN WANG, FEIYU CHEN, LILI TANG

**Affiliations:** Department of Breast Surgery, Xiangya Hospital, Central South University, Changsha, Hunan 410008, P.R. China

**Keywords:** breast cancer, inflammation, interleukin-32

## Abstract

Interleukin (IL)-32 is a newly identified cytokine in humans and primates. It has been established that IL-32 may antagonize cancer growth. However, to the best of our knowledge, the direct effect of IL-32 on breast cancer cell growth has not yet been investigated. In addition, rodents lack the expression of IL-32; hence, the effects of IL-32 on breast cancer xenografts in nude mice have not been studied. The present study aimed to examine the potential regulatory effects of IL-32 on breast cancer cells in nude mice. The effects of IL-32 on tumor cell growth in cell cuture and a tumor xenograft model were investigated, as well as the effects of IL-32 on apoptosis. The effects of IL-32 on cell proliferation and apoptosis were investigated by MTT assay and TUNEL staining, respectively. The results revealed that IL-32 increases the proliferation rate of cancer cells and decreases the rate of apoptosis, In addition, IL-32 was found to enhance the growth of tumor xenografts *in vivo*. In summary, IL-32 may represent a useful therapeutic target for human breast cancer.

## Introduction

Interleukin (IL)-32, or NK4, is a novel cytokine which was originally isolated from activated T cells. Treatment of lymphocytes with IL-32 may induce the expression of different cytokines, including tumor necrosis factor (TNF)-α or IL-8, which have been found to be involved in multiple inflammatory processes and cancer progression. Notably, a gene homolog of IL-32 was not found in rodents, hence previous study of this cytokine has been limited to cell lines *in vitro*.

Breast cancer represents a malignant cancer with serious consequences for the female population (1–3). There have been >400,000 breast cancer related mortalities per year in the pastdecade (4–6). Early diagnosis represents the most important factor for successful treatment in the early stages; while the prognosis for patients diagnosed in the later stages is notably poorer (1,2,7,8). The current efforts in treating breast cancer focus on multiple signaling pathways, which are involved in tumor formation, growth and metastasis (9–11).

The present study aimed to examine the potential effects of IL-32 on the development and progression of breast cancer. Cultured breast cancer cell lines and a tumor graft model were employed to assist in understanding how this inflammatory cytokine contributes to the growth and survival of tumor cells *in vitro* and *in vivo*.

## Materials and methods

### Ethics

This animal study has been approved by the Animal Research Ethics Committee of Xiangya Hospital, Central South University (Changsha, China). All procedures requiring animal manipulation followed the animal experiment guidelines of the Animal Center of Central South University. The approval number of this project is CSU3009-2011-BC009.

### Agents and cell culture

IL-32 was obtained from He’nuo Biotech (Changsha, China). The MCF-7 cell line was purchased from Gongji Biotech (Shanghai, China), and cultured in Dulbecco’s modified Eagle’s medium (DMEM; Invitrogen Life Technologies, Carlsbad, CA, USA) with 100 mg/ml penicillin (Sigma Corporation, Shanghai, China) and 10% fetal bovine serum (Sigma Corporation) at 37°C in 5% CO_2_. For 12 h glucose withdrawl, the glucose was removed from the medium for 12 h, with all other components maintained. At the end of the withdrawl period, the glucose-containing medium was used again.

### 3-(4, 5-dimethylthiazol-2-yl)-2,5-diphenyltetrazolium bromide (MTT) assay

In order to examine the cell viability, an MTT assay was performed. A total of 6,000 cells were seeded into 96-well plates and cultured for 24 or 48 h in the presence of IL-32 at 10, 100 or 500 ng/ml.

Following incubation for 24 or 48 h, 150 μl 5 mg/ml MTT solution was added for 2 h. The supernatant was removed following centrifugation at 5,000 × g for 2 min at 4°C and 150 μl DMSO was added to each well for absorbance reading at a wavelength of 490 nm using a plate reader (Bio-Rad 680; Bio-Rad Laboratories, Shanghai, China). The assay was repeated six times.

### Caspase-3 assay and TUNEL staining

In order to measure the levels of cell apoptosis, the Caspase-3 activity assay (Roche Diagnostics, Indianapolis, IN, USA) was used. Additionally, the TUNEL kit (Roche Diagnostics) was adopted for staining and cell counting, according to the manufacturer’s instructions. Following labeling with fluorescein isothiocyanate, the positive cells were counted under a Zeiss Axio Imager 2 microscope (Zeiss, Oberkochen, Germany; magnification, ×40; 10 sites from each experiment, repeated 3 times).

### Animal study

For tumor graft formation, the MDA-MB-231 estrogen-independent breast cancer cell line (Gongji Biotech, Shanghai, China) was employed as previously described (12). In total, 2×10^7^ MDA-MB-231 cells were separated with 170 μl DMEM medium and inoculated subcutaneously into the right flanks of 48 nude mice (weight, 20–30 g; age, 2 months; Animal Research Center, Xiangya Hospital).

For the drug treatment, 0.2 or 1 mg/kg IL-32 was administrated via careful intraperitoneal (i.p.) injection every two days. A total of 16 mice were treated with saline (control group), 16 were treated with 0.2 mg/kg IL-32 and 16 were treated with 1 mg/kg IL-32. An insulin injection syringe (total volume, 0.5 ml; Xiangya Hospital) was used to prevent any harm caused by normal syringe needles.

Tumor size was measured every 5 days for 30 days and the volume was calculated as follows: Volume = 0.5 multiplied by length multiplied by width multiplied by width. On day 30, the mice were sacrificed by CO_2_ inhalation and the tumors were harvested and fixed in 10% formalin. The tumors were then cut into 10 μm sections in a Cryostat (Leica CM 3050S; Leica Microsystems, Wetzlar, Germany). A TUNEL *in situ* cell deah dection kit (Roche Diagostics) was used for staining and cell counting, according to the manufacturer’s instructions. Briefly, the cells were incubated with DNA-labeling solution for 60 min at 37°C, then washed three times with rinse buffer prior to incubation with the antibody-staining solution for 30 min at room temperature in the dark.

### Statistics

Data are presented as the mean ± standard deviation and were analyzed using SPSS software, version 11.0 (SPSS, Inc., Chicago, IL, USA). A t*-*test and analysis of variance were used to compare differences between the groups. P<0.05 was considered to indicate a statistically significant difference.

## Results

### IL-32 increases cell proliferation

The results demonstrated that IL-32 treatment reduced the cell numbers compared with the untreated cells, as detected by light absorbance. After 24 or 48 h, the number of viable cells significantly increased following 100 or 500 ng/ml IL-32 treatment (P<0.01). In addition, this association appeared to be concentration-dependent (Table I).

### IL-32 decreases the levels of cancer cell apoptosis in vitro following glucose withdrawal

Given that IL-32 treatment increased cell proliferation, the subsequent step was to investigate whether this treatment leads to a reduction in cell apoptosis after 12 h glucose withdrawal. Using TUNEL staining, the rate of apoptosis in the control group was revealed to be 56.7±8.9%, while those in the 10, 100 and 500 ng/ml IL-32-treated groups were 44.2±6.7% (P<0.01), 37.7±6.9% (P<0.01) and 34.3±5.1% (P<0.01) at the 24 h time point (12 h following the induction of glucose withdrawal).

### IL-32 increases the growth of tumor xenografts

**Figure 1:**
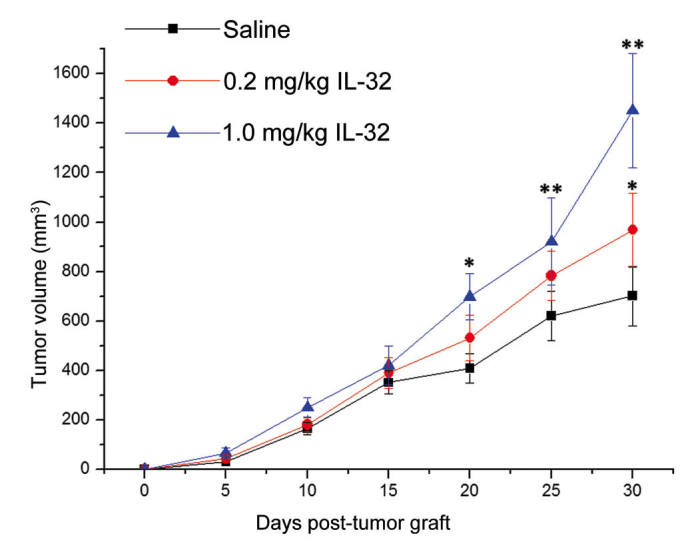
Interleukin (IL)-32 enhances the growth of breast cancer cell xenografts in nude mice. ^*^P<0.05 and ^**^P<0.01, compared with the control group.

Following the results of the cell proliferation assay, it was investigated whether IL-32 contributed to tumor growth *in vivo*. It was revealed that IL-32 treatment at 0.2 mg/kg and 1 mg/kg increased the tumor graft growth compared with treatment with saline only (Fig. 1).

## Discussion

To the best of our knowledge, the current study is the first to characterize the effects of IL-32 on cancer cells directly; specifically, on breast cancer cells. IL-32 showed cancer-promoting effects *in vitro* and *in vivo*. These results are consistent with previous studies showing the involvement of IL-32 in response to certain inflammatory processes (13–15). IL-32 may act as an intracellular controller, determining the cell survival and death (13). Indeed, the current study found that in culture IL-32 seems to enhance survival of breast cancer cells in a glucose-withdrawn environment. Additionally, IL-32 increased the total number of total, as revealed by increased cell proliferation, which is consistent with a previous finding showing that IL-32 induces the expansion of hematopoietic progenitor cells (16).

It should be noted that in a previous study, the IL-32γ variant has been shown to inhibit cancer growth by silencing the NF-κB and STAT3 signaling pathways (17). In the aforementioned study, colon cancer cells were directly transfected with IL-32γ, prior to transplantation in nude mice. The present study used an i.p. injection of IL-32, therefore systemic IL-32 may trigger additional effects, for example the recruitment of other inflammatory signaling pathways. We hypothesized that since cancer tissues have an increased expression of IL-32, this indicated the potential involvement of this cytokine in cancer growth, for example in pancreatic cancer (18,19).

In conclusion, the results of the current study suggest that IL-32 exerts modulatory effects on the growth and survival of breast cancer cells. Therefore IL-32 may be a novel therapeutic target for breast cancer, and potentially other types of cancer as well. Future studies, which investigate the combination of IL-32 silencing and chemotherapy drugs for breast cancer treatment may be useful.

## Figures and Tables

**Table I:** Effect of interleukin (IL)-32 on cell proliferation.

	24 h				48 h			
		
		IL-32				IL-32		
				
	Control	10 ng/ml	100 ng/ml	500 ng/ml	Control	10 ng/ml	100 ng/ml	500 ng/ml
Viable cells (%)	100 (normalized)	106.2±4.4	130.1±7.2^b^	144.6±5.3^b^	100 (normalized)	108.9±5.3^a^	134.7±7.6^b^	157.2±7.4^b^

a P<0.05 and

b P<0.01, compared with the control group.
